# Co-treatment of β-carotene with acetamiprid provides protection against acetamiprid induced hepatic and renal toxicity via modulation of the antioxidant system

**DOI:** 10.1186/s40360-025-00953-9

**Published:** 2025-06-23

**Authors:** Eatemad A. Awadalla, Yahia A. Amin, Rana A. Ali, Samia A. Gbr, Wafaa Ibraheem Gelany, Amna H. M. Nour

**Affiliations:** 1 Zoology Department, Faculty of Science, Aswan University, Aswan, 81528 Egypt; 2 Department of Theriogenology, Faculty of Veterinary Medicine, Aswan University, Aswan, 81528 Egypt; 3 Zoology Department, Faculty of Science, South Valley University, Qena, 83523 Egypt

**Keywords:** Acetamiprid, Β-carotene, Liver toxicity, Kidney toxicity, Total oxidative stress, Total antioxidant capacity

## Abstract

**Background:**

Acetamiprid (ACMP), one of the most widely used and effective insecticides globally, can pose potential toxicity to mammals. β-carotene (βC) is a prominent carotenoid precursor to vitamin A and exhibits antioxidant properties. This study evaluated the protective effect of βC as an antioxidant against ACMP toxicity in rats.

**Methods:**

A total of 40 male albino rats were divided into four groups: the control group received 1% DMSO; the βC group received 100 mg/kg of β-carotene; the ACMP group received 40 mg/kg of acetamiprid; and the ACMP + βC group received both ACMP and βC. Liver and kidney tissues were used for biochemical analyses (total oxidative stress [TOS] and total antioxidant capacity [TAC]), as well as histopathological, histochemical, and immunohistochemical analyses (MPO immunostaining).

**Results:**

The ACMP group, compared to the control and βC groups, showed a significant increase in TOS levels (*p* < 0.001) in both liver and kidney tissue homogenates, along with a significant decrease in TAC in the same tissues. The ACMP + βC group exhibited significantly lower TOS levels (*p* < 0.01) and significantly higher TAC levels (*p* < 0.05) than the ACMP group in the liver and kidney tissue homogenates. Furthermore, histopathological alterations were observed in both organs. Changes such as congestion of central veins and blood sinusoids in the liver were noted. In most cases, hepatocytes exhibited basophilic cytoplasm, vacuolar cytoplasm, and pyknotic nuclei. Renal alterations included atrophy of the renal corpuscle, reduced glomerular cellularity, marked dilation of the urinary space, desquamated epithelial cells in the tubular lumen, inflammatory cell infiltration, and congestion of interstitial blood capillaries. In contrast, the ACMP + βC group showed significant improvements in these histopathological changes. MPO immunostaining revealed a significant increase in the ACMP group compared to the other three groups.

**Conclusion:**

Co-treatment with β-carotene and acetamiprid reduced ACMP-induced toxicity by enhancing antioxidant capacity and reducing oxidative stress.

## Background

Neonicotinoids are a class of synthetic insecticides widely used in agriculture to protect crops from insect infestations [1, 2]. These insecticides have been extensively applied worldwide due to their potent effectiveness against insects and other pests. However, even when used in minimal doses, they tend to persist in the environment because of the high volume of their use [3]. Acetamiprid (ACMP) is a neonicotinoid insecticide and one of the most effective insecticides globally for crop protection and controlling fleas in livestock and pets. However, it poses potential toxicity to mammals. As ACMP is water-soluble and rapidly absorbed by plants and animals, it is metabolized in the liver before being excreted by the kidneys [4]. The liver, a key organ for detoxification, is vulnerable to various disorders due to exposure to environmental pollutants. The kidneys, which share the responsibility for excretion with the liver, play a significant role in detoxifying toxic chemicals. ACMP is primarily excreted in urine. Due to its water solubility and low molecular weight, it can easily pass through the glomerulus [5].

Acetamiprid has deleterious effects on the liver and kidneys, potentially due to oxidative stress caused by its metabolites and its harmful effects on lipids [6]. Administration of ACMP leads to decreased antioxidant status and increased oxidative stress [2]. Additionally, ACMP administration causes significant oxidative damage in the liver, kidneys, and brain [7, 8]. Furthermore, previous studies have shown that ACMP induces several histopathological changes in the heart, brain, and testes [4, 9].

Today, the scientific community is focused on the role of oxidative stress in the initiation and development of various complications and the role of antioxidants in reducing and preventing these deleterious effects [10–16]. One prominent antioxidant is β-carotene (βC), a bright red-orange pigment naturally occurring in plant-based foods such as carrots, tomatoes, potatoes, watermelon, and many others. β-carotene is an antioxidant and a colorant in food products [17]. Furthermore, it is recognized for its anticancer properties [18] and anti-inflammatory effects [19]. The Food and Drug Administration (FDA) has classified βC as generally safe and recommends its use as a nutrient supplement for adult and infant foods [20]. Therefore, βC is widely employed in preventing and treating disorders caused by oxidative stress [21].

Although βC is one of the prominent antioxidants, some limitations have been reported. The European Food Safety Authority (EFSA) advises smokers to avoid consuming food supplements containing βC. Additionally, the use of supplemental β-carotene by the general population should be limited to meeting vitamin A requirements. However, this conclusion does not apply to the potential use of supplemental βC for therapeutic purposes under medical supervision (e.g., as a source of pro-vitamin A in vitamin A deficiency or for treating erythropoietic protoporphyria) [22].

While previous studies have documented the toxic effects of ACMP and the general antioxidant properties of βC, to the best of our knowledge, no studies have specifically investigated the protective role of βC against acetamiprid-induced toxicity. The present work is the first to comprehensively assess the co-treatment effects of βC on acetamiprid-induced liver and kidney damage in vivo. This study highlights a potential therapeutic approach to mitigating the harmful effects of pesticide exposure in non-target organisms through nutritional antioxidants such as βC. Our findings contribute to a broader understanding of how naturally occurring antioxidants may be leveraged to counteract environmental toxicants and may support future research in ecotoxicology and preventive medicine. This study aimed to investigate the protective effects of β-carotene against hepatic and renal toxicity induced by ACMP, a neonicotinoid pesticide known to exert oxidative stress-related toxic effects. Specifically, we sought to determine whether co-treatment with β-carotene could alleviate this toxicity by modulating the antioxidant defense system in exposed rats.

## Materials and methods

### Materials

βC was purchased from Sigma-Aldrich Co. (USA), with CAS number 7235-40-7. Acetamiprid was obtained from Aqua Chemical Ltd. (Japan), with lot number GC20081209C. Commercial kits for total oxidative stress (TOS) were supplied by Diatechnology, Egypt, with catalog number BC-2022. Commercial kits for total antioxidant capacity (TAC) and total protein (TP) were purchased from Bio Diagnostics (Egypt), with catalog numbers TA 2513 and TP 2020, respectively. Primary and secondary antibody kits were obtained from Abclonal Technology Co. (USA), with catalog numbers A23937 and AS014, respectively. All other chemicals used were of the highest quality.

### Ethical statement

The Animal Ethics Committee of South Valley University in Qena, Egypt, approved all experimental methods, which were conducted in compliance with regional institutional policies (code number: 003/12/22).

### Animals and experimental design

In the present study, 40 male albino Sprague-Dawley (SD) rats (weighing 140 ± 20 g) were obtained from the Animal House of the Department of Zoology, Faculty of Science, South Valley University, Qena, Egypt. Male rats were chosen to minimize the potential confounding effects of hormonal fluctuations associated with the estrous cycle in females, which can influence metabolic, oxidative stress, and toxicological responses to chemical exposure. This approach allows for greater consistency and reproducibility in the measured outcomes. Moreover, using male animals in toxicological studies is a widely accepted initial step to establish baseline effects before expanding investigations to include both sexes. Additionally, male rats are often preferred in research due to their more consistent physiological responses compared to females, who can exhibit variability due to hormonal cycles [23]. The rats were housed in wire mesh cages in a controlled environment with a 12-hour light/dark cycle, 23 ± 2 °C temperature, and 55% relative humidity. They were acclimatized for one week under standard laboratory conditions and given free access to standard commercial pellets and water ad libitum. In the second week, the rats were randomly divided into four groups, with ten in each group.

All treatments were administered orally via intragastric intubation daily for 30 days. Acetamiprid was dissolved in 1% dimethyl sulfoxide (DMSO). The groups were assigned as follows: Group I (control) received 1% DMSO [24]; Group II (βC group) received βC (100 mg/kg) [25]; Group III (ACMP group) received acetamiprid (40 mg/kg) [26]; and Group IV (ACMP + βC group) received βC (100 mg/kg), followed by acetamiprid (40 mg/kg) after 30 min. Throughout the experimental period, all animals were observed daily for general signs of toxicity, including changes in behavior, posture, locomotor activity, skin and eye conditions, and food and water intake. Mortality was also monitored throughout the study.

Twenty-four hours after receiving the final dose, animals were anesthetized and then killed by cervical decapitation. After rapid dissection, the liver and kidneys were removed quickly and gently cut into small pieces, which were frozen at -80 °C for biochemical evaluation. Another portion of the selected organs was rapidly transferred into 10% buffered formalin for further processing.

### Total oxidative status and total antioxidant capacity assays of tissue homogenates

The frozen fragments of the liver and kidney were homogenized using phosphate buffer (pH 7.4) with a tissue homogenizer (Virtiz T-25 Polytron). The homogenates were then centrifuged at 4000 rpm for 30 min at 4 °C. The supernatants from the tissue homogenates were used for colorimetric tests of total oxidative status (TOS) and total antioxidant capacity (TAC) using a Shimadzu UV-visible spectrophotometer (UVmini-1240, Shimadzu Corporation, Japan). Prior to these tests, the total protein content of each tissue homogenate sample was determined spectrophotometrically. Each tissue homogenate sample’s TOS and TAC were calculated by dividing the corresponding amounts by the total protein content (mmol/g tissue protein) [27].

### Histological and histochemical examinations

After fixation in 10% neutral buffered formalin (pH 7.2), liver and kidney samples were dehydrated in an escalating series of ethanol, cleaned in methyl benzoate, and embedded in paraffin wax. The paraffin blocks were sectioned using a microtome at a thickness of 5–6 μm. The tissue sections were then deparaffinized and rehydrated to prepare them for staining with various dyes, including Harris’s hematoxylin and eosin (H&E), Masson’s trichrome stain, Periodic Acid-Schiff (PAS), and an immunohistochemistry staining protocol. H&E staining was used to investigate the general tissue structure, Masson’s trichrome stain was employed to assess collagenous fibers, and the PAS technique was applied to examine general carbohydrate content in the selected tissues. Additionally, immunohistochemistry was performed to evaluate the expression of myeloperoxidase (MPO).

### Hematoxylin and Eosin (H&E) staining method

The sections were stained with Harris’s hematoxylin solution for 5–10 min to visualize nuclei, followed by rinsing in tap water. Differentiation was performed using acid alcohol (1% HCl in 70% ethanol) for a few seconds to remove excess hematoxylin. After rinsing, sections were “blued” in alkaline tap water or lithium carbonate solution to enhance nuclear staining. Subsequently, sections were counterstained with eosin for 1–2 min to highlight cytoplasmic and extracellular components. After dehydration through ascending grades of ethanol and clearing in xylene, sections were mounted with a synthetic resin for microscopic examination [28].

### Masson’s trichrome staining method

The sections were stained in Weigert’s iron hematoxylin solution to visualize nuclei, followed by rinsing in running tap water. Subsequently, tissues were stained with Biebrich scarlet-acid fuchsin solution to stain cytoplasm and muscle fibers. Differentiation was performed using phosphomolybdic-phosphotungstic acid solution to remove excess stain, and collagen fibers were then selectively stained using aniline blue. Sections were briefly treated with acetic acid to enhance color differentiation, dehydrated through ascending alcohols, cleared in xylene, and mounted with a resinous medium for microscopic evaluation of collagen fiber distribution and density [28].

### Periodic Acid-Schiff (PAS) staining method

The sections were oxidized in 0.5–1% periodic acid solution for 5–10 min to generate aldehyde groups from tissue carbohydrates. After rinsing in distilled water, the sections were treated with Schiff’s reagent for 10–20 min to visualize the aldehyde groups as a magenta coloration. Following another rinse in running tap water to develop the color fully, sections were counterstained lightly with hematoxylin to visualize nuclei. Finally, tissues were dehydrated through ascending ethanol series, cleared in xylene, and mounted with a resinous medium for microscopic examination of polysaccharide and glycoprotein distribution [28].

### Immunohistochemistry staining protocol

Tissue sections of 4–5 μm thickness were cut from formalin-fixed, paraffin-embedded liver and kidney samples. The sections were deparaffinized and rehydrated. Myeloperoxidase expression in both liver and kidney tissues was demonstrated using the ABclonal kit. Antigen retrieval solution was applied, followed by inactivation of endogenous peroxidase with 3% hydrogen peroxide for 15 min. A blocking solution was then applied for 1 h. The sections were incubated overnight with rabbit monoclonal primary antibody (Catalog No. A23937) in a humidified chamber at 4 °C. After primary antibody incubation, a peroxidase-labeled secondary antibody [HRP goat-anti-rabbit IgG (H + L), Catalog No. AS014] was applied at a 1:200 dilution and incubated for 30 min. Freshly prepared DAB substrate and chromogen were applied at room temperature for 2 to 5 min. Finally, the sections were counterstained with hematoxylin, dehydrated, and mounted.

Histopathological and immunological alterations were examined under a high-power light microscope (Olympus BX43F, Tokyo, Japan). Image analysis was performed using a personal computer, camera, and software (Olympus DP74, Tokyo, Japan) and an optical microscope at the Zoology Department, Faculty of Science, Aswan University.

Tissue damage was evaluated using a standardized histopathological scoring system. The scoring criteria included cellular degeneration, inflammation, necrosis, and structural alterations, following the methods established by Sanz-Nogués et al. [29].

### Morphometric analysis

Morphometric analysis was used to quantify structural changes identified through histological analysis of liver and kidney tissue. After histological processing of rat livers and kidneys, digital images were captured at 40X magnification using a digital camera connected to a light microscope. Morphometric analysis was performed using the ImageJ computerized image analysis software system, version 6. Spatial calibration with an object micrometer was carried out before each analysis. Five images were selected from each animal in each group. The following morphometric parameters were measured in the liver and kidney tissues: %collagen fiber intensity/surface area, %polysaccharide content/surface area, and %MPO-positive reaction intensity/surface area.

### Statistical analysis

All data from the biochemical and morphometric analyses were analyzed using Prism 6.0 software (GraphPad Software, Inc., San Diego, USA) and Microsoft Excel. The results were expressed as means ± standard error (SE). One-way analysis of variance (ANOVA) was used to test for differences between the means, followed by the Student-Newman-Keuls test for multiple comparisons. Statistical significance was set at *p* < 0.05.

## Results

### General toxicity effect

Table 1 shows the general toxicity profile of acetamiprid. The results indicated no mortality was recorded. The general toxicity effects included decreased food intake, associated with marked irritation and excitement in the early stages. In the later stages, there was a decrease in motor activity, a tendency toward isolation (remaining in a corner), and skin and eye irritation. Finally, general weakness was observed over time. Histopathological changed were observed in the liver and kidney tissues.

### Total oxidative stress and total antioxidant capacity in the liver and kidney

Table 2 shows no significant difference in the mean hepatic and renal homogenate levels of TOS and TAC (each *p* > 0.05) in the βC-treated group compared with the control group. In contrast, the ACMP group exhibited highly significant levels of TOS (mmol/g tissue protein) (*p* < 0.001) and significantly lower levels of TAC in the hepatic (*p* < 0.05) and renal (*p* < 0.001) homogenates compared to the control group. However, in the ACMP + βC group, both hepatic and renal homogenates showed a significant decrease in TOS (each *p* < 0.01) and a significantly higher level of TAC (each *p* < 0.05) compared to the ACMP group.

### Hematoxylin and Eosin staining of the liver

**Fig. 1:**
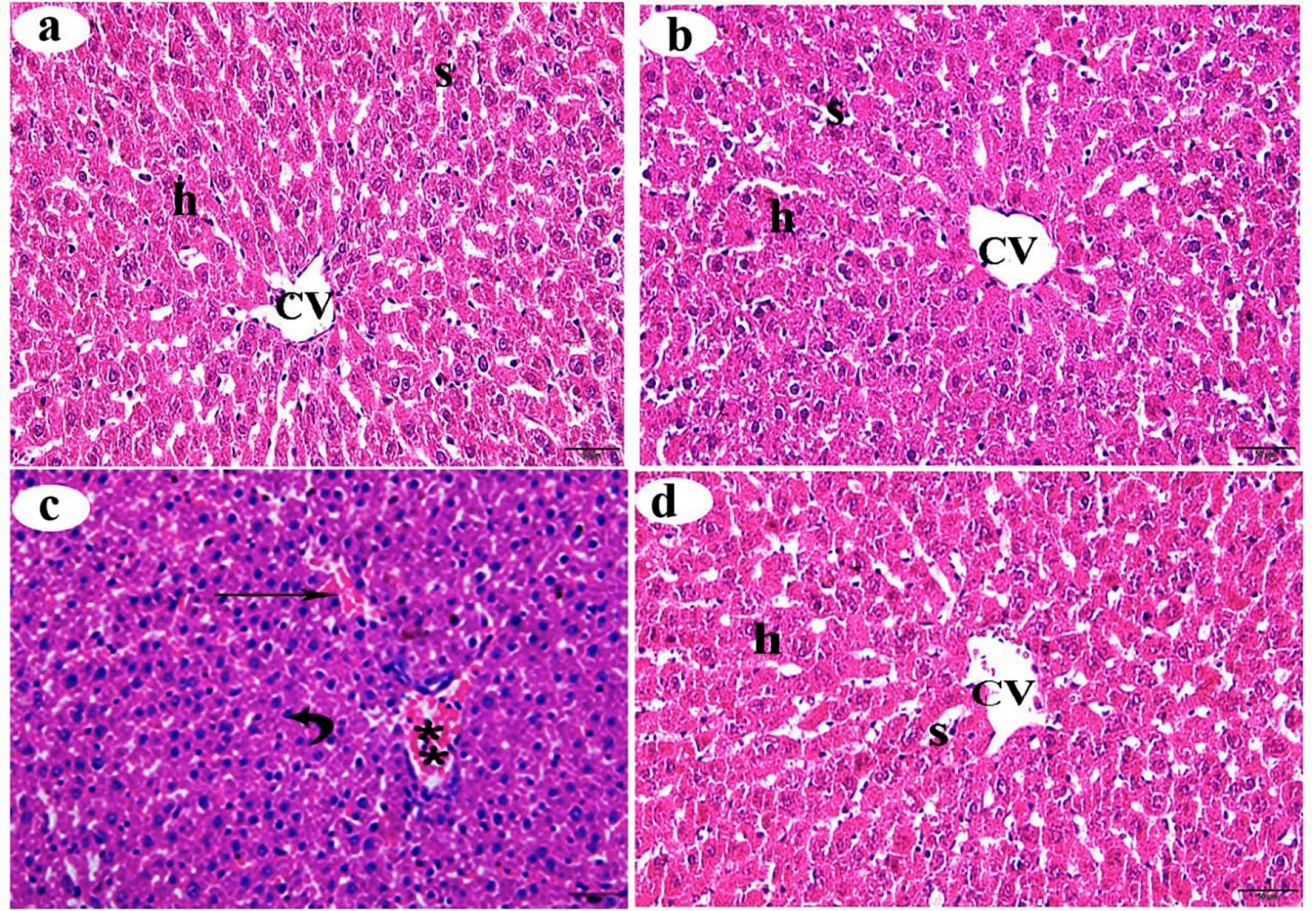
Histological alterations (stained with H&E) of the centrilobular zone of the liver obtained from adult male albino rats after oral administration of ACMP, βC, and/or their combination (ACMP + βC) compared to control rats. (**a**) Control group; (**b**) βC-treated group; (**c**) ACMP-treated group; and (**d**) ACMP + βC group. h, hepatocytes; CV, central vein; S, hepatic sinusoids; stars, blood congestion of ventral vein; curved arrow shows pyknotic nuclei; thin arrows show blood congestion of hepatic sinusoids; βC, β-Carotene; ACMP, Acetamiprid; H&E, hematoxylin and eosin (Original magnification: a-d, X400)

Histological examination of H&E-stained liver sections from the control and βC-treated groups revealed a normal histological structure, including the typical arrangement of hepatic cords, central veins, and hepatic sinusoids, which extend between the hepatic cords (Figs. 1a and b, respectively). In contrast, various histological changes were observed in the liver of the ACMP-treated group, such as congestion of the central veins and blood sinusoids. The hepatocytes exhibited basophilic cytoplasm, some displayed vacuolar cytoplasm, and the majority had pyknotic nuclei (Fig. 1c). The liver section from the ACMP + βC group showed a reduction in these degenerative changes and appeared to have a structure similar to that of the control group, to a significant degree (Fig. 1d). Table 3 summarizes the histopathological changes recorded in the liver of the studied groups.

### Masson’s trichrome staining of the liver

**Fig. 2:**
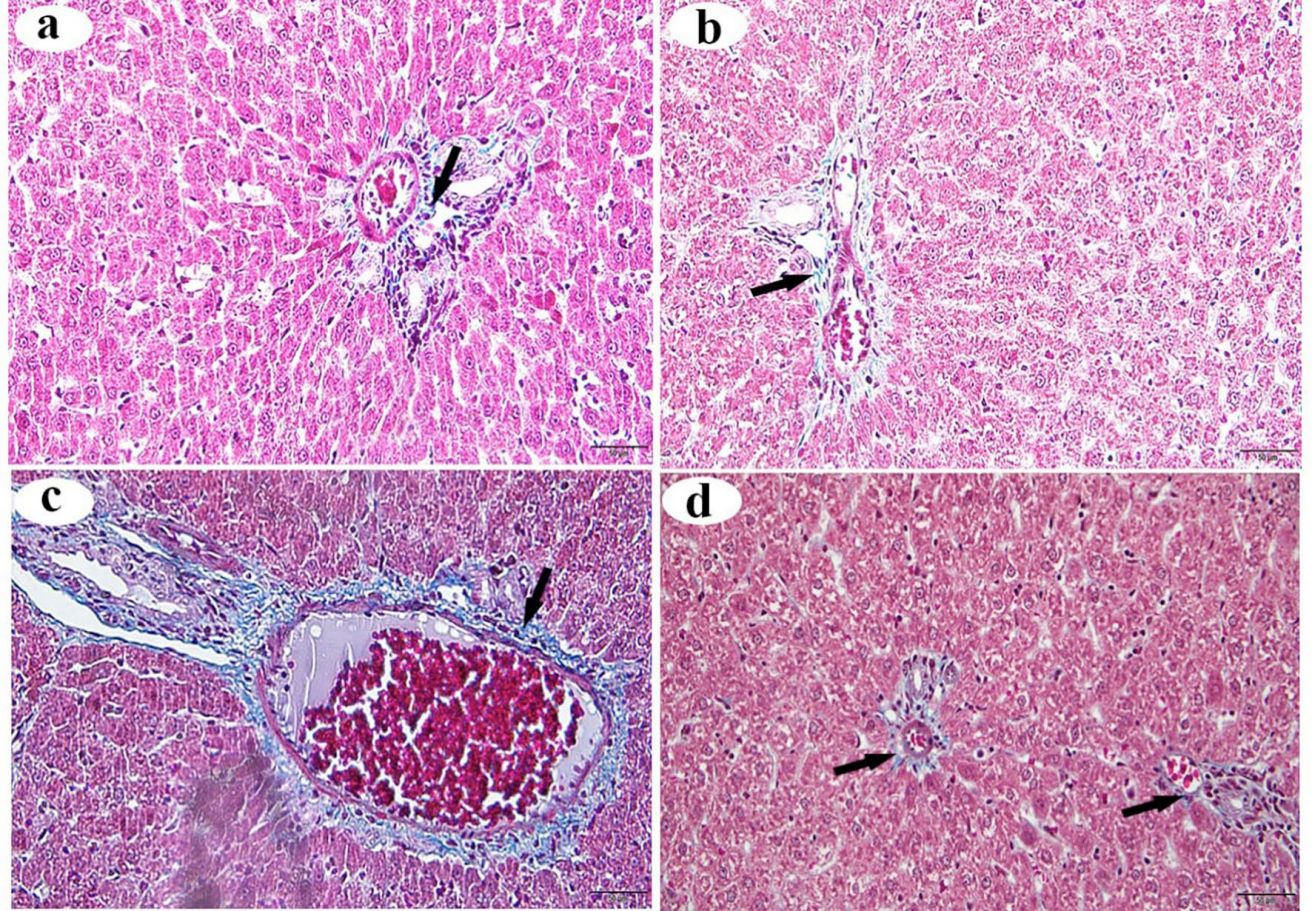
Histological alterations (stained with Masson’s trichrome stain) of the portal area of the liver obtained from adult male albino rats after oral administration of ACMP, βC, and/or their combination (ACMP + βC) compared to control rats. (**a**) Control group; (**b**) βC-treated group; (**c**) ACMP-treated group; and (**d**) ACMP + βC group. The arrow shows the distribution of collagen fibers at the portal area (Original magnification: a-d, X400)

Blue-colored stripes of sparse collagen fibers were observed around the central vein in the control and βC-treated group sections (Figs. 2a and b, respectively). In contrast, the ACMP group sections exhibited a pronounced increase in the thickness of collagen fibers surrounding the congested central veins (Fig. 2c). The ACMP + βC group showed a small amount of dispersed collagen fibers around the central veins (Fig. 2d).

### Morphometric analysis

Morphometric analysis of collagen fiber intensity revealed a highly significant increase (*p* < 0.001) in the ACMP group compared with the control and βC-treated groups. When compared with the ACMP group, the morphometric intensity of collagen fibers in the ACMP + βC group significantly decreased (*p* < 0.001); however, no significant difference was observed between the ACMP + βC group and the control group (*p* > 0.05) (Table 4).

### Histochemical changes in the liver

**Fig. 3:**
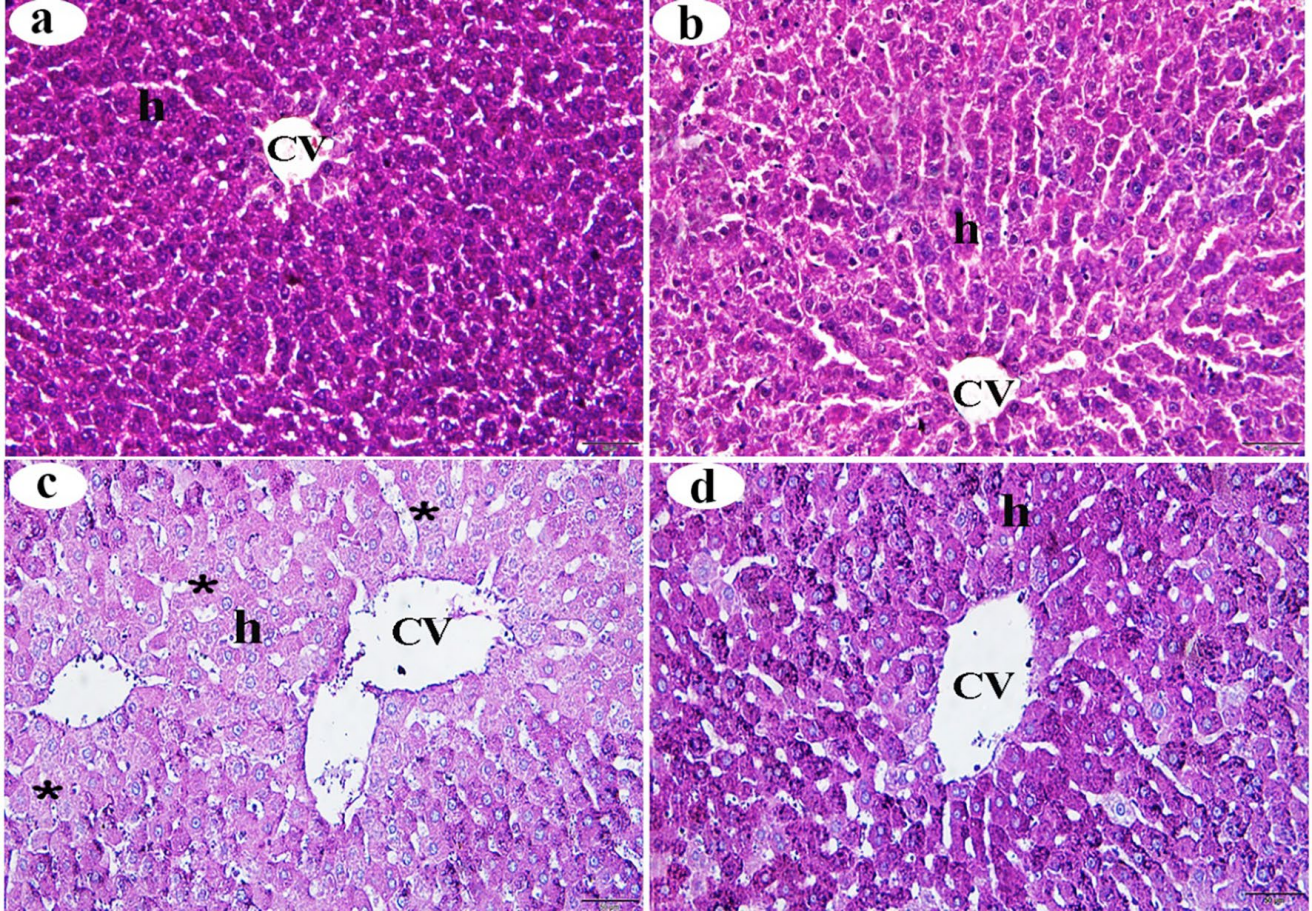
Histochemical changes (stained with PAS-reaction) of the centrilobular zone of the liver obtained from adult male albino rats after oral administration of ACMP, βC, and/or their combination (ACMP + βC) compared to control rats. (**a**) Control group; (**b**) βC-treated group; (**c**) ACMP-treated group; and (**d**) ACMP + βC group. h, hepatocytes; CV, central vein; stars show a decrease in the carbohydrate content of the majority of hepatocytes; PAS, periodic acid–Schiff (Original magnification: a-d, X400)

PAS staining revealed the presence of a considerable amount of glycogen granules in the hepatocytes of both the control and βC-treated groups (Figs. 3a and b, respectively). Compared with the control group, a decrease in the PAS reaction was observed in the liver tissue of the ACMP group (Fig. 3c). Furthermore, compared to the ACMP group, an increase in polysaccharide content was observed in the hepatic cells of the ACMP + βC group (Fig. 3d), which appeared similar to that in the control group. Morphometric analysis of PAS reaction intensity in the ACMP group revealed a highly significant decrease (*p* < 0.001) compared with the control group. However, the morphometric analysis of PAS reaction intensity in the ACMP + βC group showed a significant increase (*p* < 0.01) compared with the ACMP group, with no significant difference observed between the ACMP + βC group and the control group (*p* > 0.05) (Table 4).

### MPO immunostaining of the liver

**Fig. 4:**
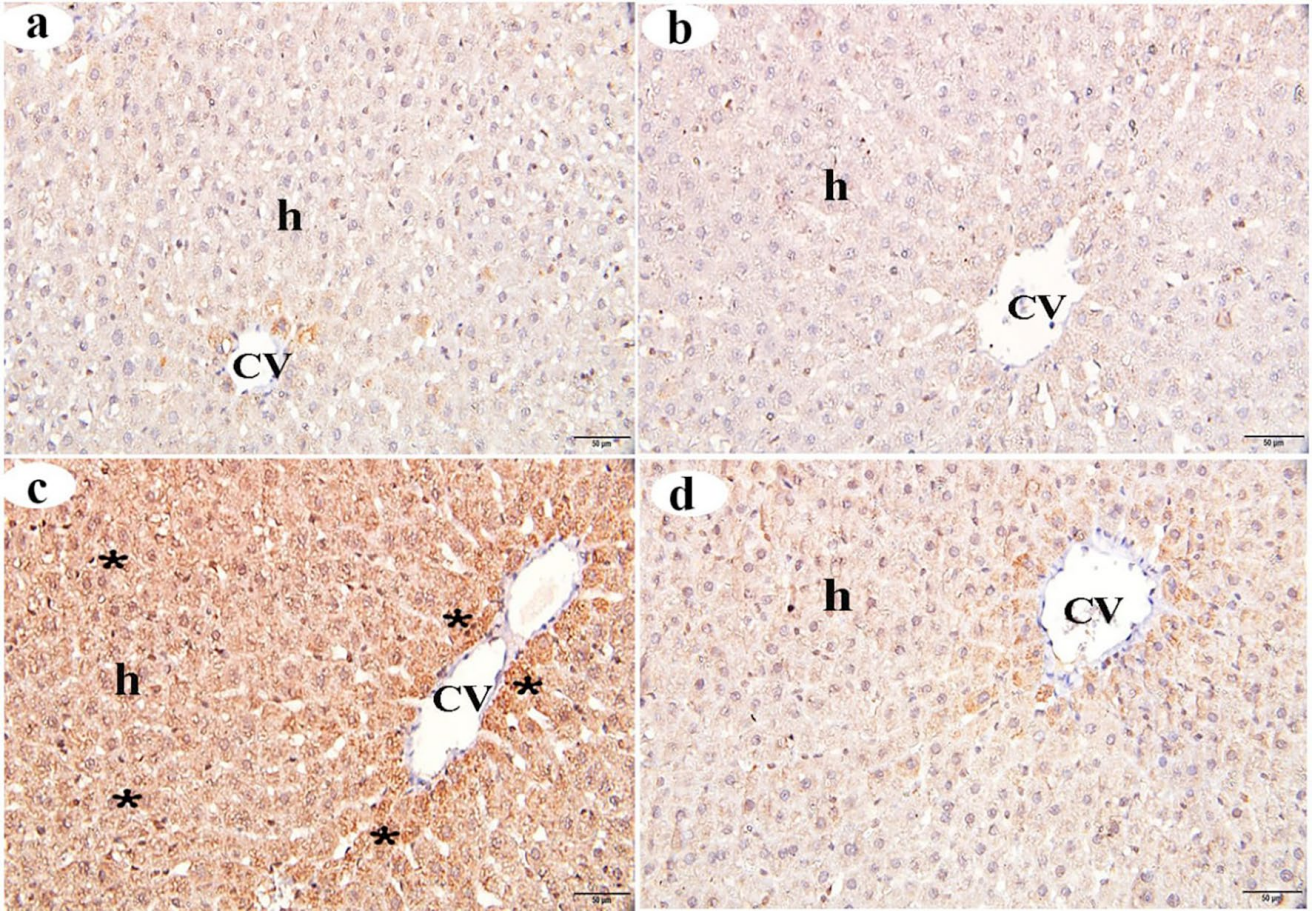
Immunohistochemical changes (stained with MPO immune-staining) of the centrilobular zone of the liver obtained from adult male albino rats after oral administration of ACMP, βC, and/or their combination (ACMP + βC) compared to control rats. (**a**) Control group; (**b**) βC-treated group; (**c**) ACMP-treated group; and (**d**) ACMP + βC group. h, hepatocytes; CV, central vein; stars show a strong positive MPO reaction in the majority of hepatocytes; MPO, myeloperoxidase (Original magnification: a-d, X400)

MPO immunostaining was used as a marker of inflammation in hepatic tissue. Immunohistochemical staining for MPO revealed very weak MPO expression in the hepatocytes of both the control group (Fig. 4a) and the βC group (Fig. 4b). In contrast, ACMP-treated liver sections showed a strongly positive MPO reaction, marked by a dark brown color, particularly adjacent to the central vein (Fig. 4c). A non-significant increase in the intensity of MPO immunoreactivity was observed in the centrilobular zone of liver sections from the ACMP + βC group (Fig. 4d) compared to the ACMP group. Morphometric analysis of MPO immunoreactivity intensity revealed no significant difference (*p* > 0.05) between the control and βC-treated groups. In contrast, the intensity of MPO immunoreactivity in the ACMP group showed a highly significant increase (*p* < 0.001) compared to the control group. The decrease in MPO immunoreactivity intensity in liver sections from the ACMP + βC group was significant (*p* < 0.001) compared to the ACMP group (Table 4).

### Hematoxylin and Eosin staining of the kidney

**Fig. 5:**
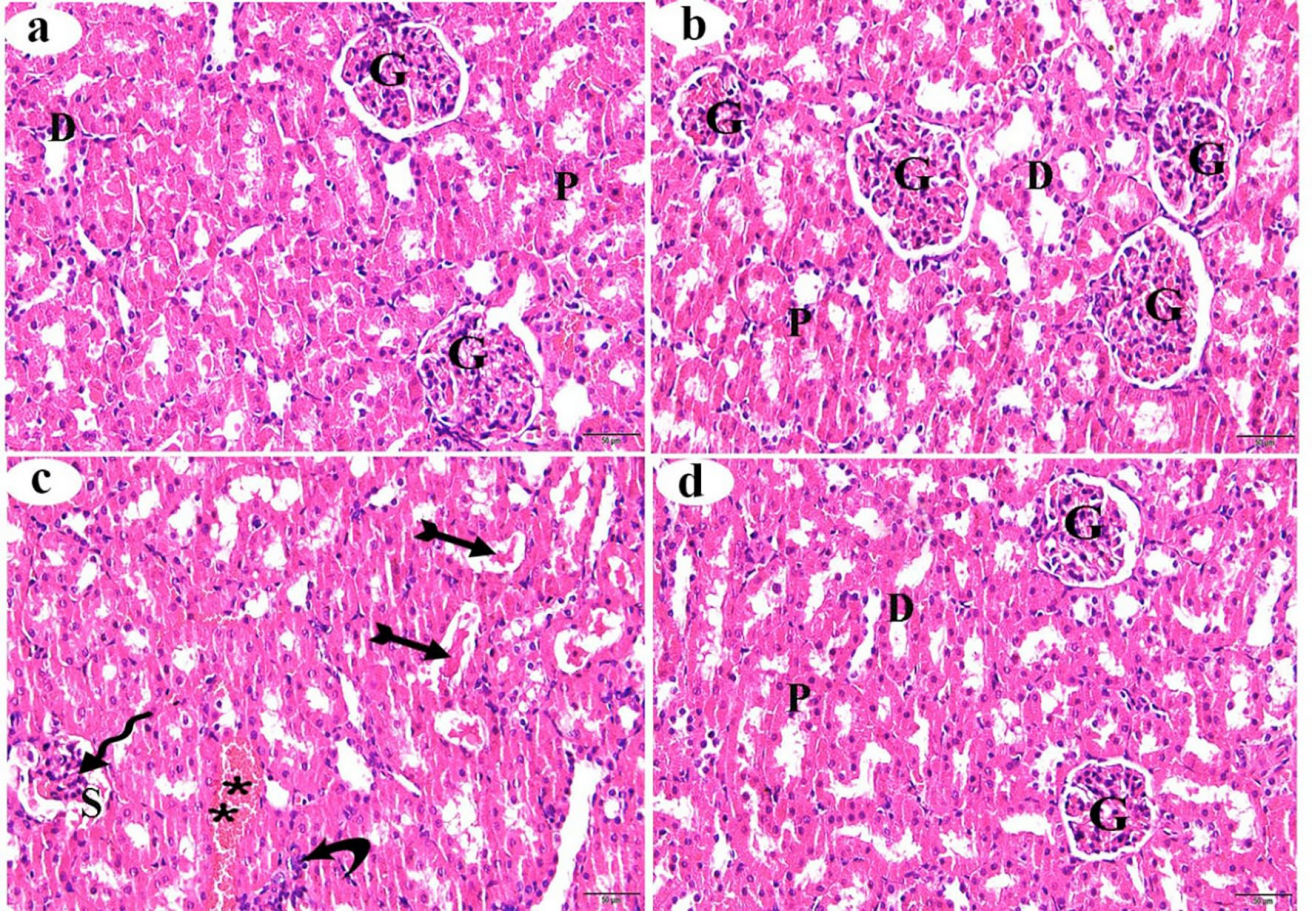
Histological alterations (stained with H&E) of the renal cortex obtained from adult male albino rats after oral administration of ACMP, βC, and/or their combination (ACMP + βC) compared to control rats. (**a**) Control group; (**b**) βC-treated group; (**c**) ACMP-treated group; and (**d**) ACMP + βC group. G, Glomerulus; P, proximal convoluted tubule; D, distal convoluted tubule; zigzag arrow shows renal corpuscle atrophy; S, marked dilation of the urinary space; forked arrows show desquamated epithelial cells in tubule lumens; curved arrow shows inflammatory cell infiltration; stars, congestion of interstitial blood capillaries; βC, β-Carotene; ACMP, Acetamiprid; H&E, hematoxylin and eosin (Original magnification: a-d, X400)

Kidney sections stained with H&E from the control and βC groups displayed normal kidney architecture with no aberrant histological alterations (Figs. 5a and b, respectively). In contrast, marked histological changes were observed in the ACMP group, including renal corpuscle atrophy, reduced glomerular cellularity, marked dilation of the urinary space, desquamated epithelial cells in the tubule lumens, inflammatory cell infiltration in the renal tissue, and congestion of interstitial blood capillaries (Fig. 5c). However, improvements in the cortical kidney architecture, including renal tubules and Malpighian corpuscles, were observed in the ACMP + βC group (Fig. 5d). The histopathological changes in the kidneys are summarized in Table 3.

### Masson’s trichrome staining of the kidney

**Fig. 6:**
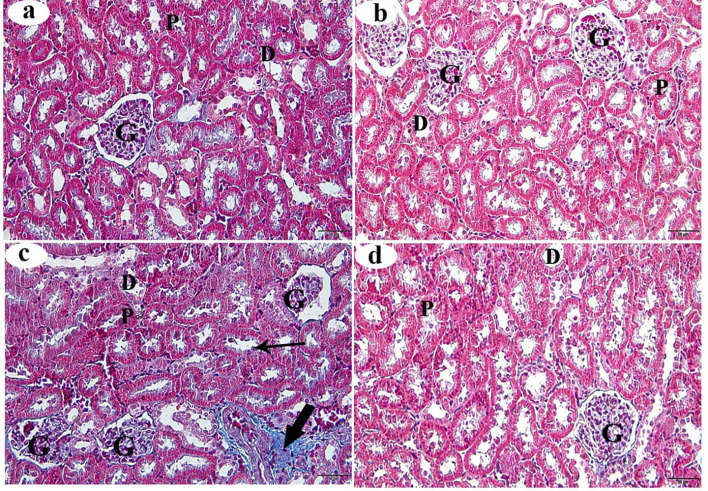
Histological alterations (stained with Masson’s trichrome stain) of the renal cortex obtained from adult male albino rats after oral administration of ACMP, βC, and/or their combination (ACMP + βC) compared to control rats. (**a**) Control group; (**b**) βC-treated group; (**c**) ACMP-treated group; and (**d**) ACMP + βC group. G, Glomerulus; P, proximal convoluted tubule; D, distal convoluted tubule; thick arrow shows an increase in collagen fibres amount; βC, β-Carotene; ACMP, Acetamiprid; H&E, hematoxylin and eosin (Original magnification: a-d, X400)

Sections from the control and βC groups stained with Masson’s trichrome stain showed a normal distribution of collagen fibers between the renal tubules and glomerular capillaries (Figs. 6a and b, respectively). In the ACMP group, an increase in collagen fiber accumulation surrounding the Bowman’s capsule, kidney tubules, and renal blood vessels was observed (Fig. 6c). Interestingly, kidney sections from the ACMP + βC group showed a noticeable reduction in collagen fibers around the renal tubules and glomerulus (Fig. 6d). Morphometric analysis of collagen fiber intensity revealed a highly significant increase (*p* < 0.001) in the ACMP group compared to the control group. The ACMP + βC group showed a significant decrease in collagen fibers (*p* < 0.001), but no significant difference was found compared with the control group (*p* > 0.05) (Table 4).

### Histochemical changes in the kidney

**Fig. 7:**
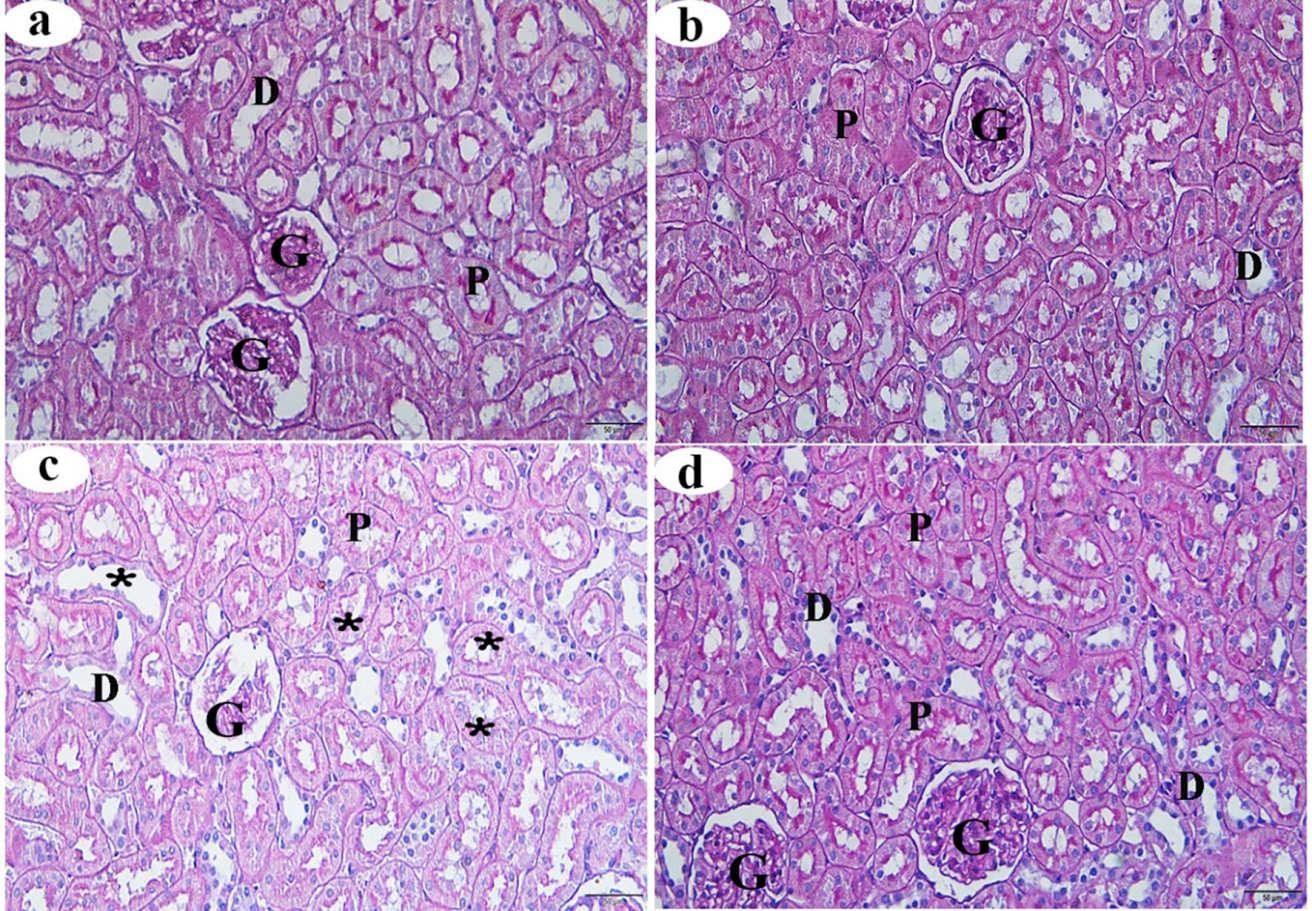
Histochemical changes (stained with PAS-reaction) of the renal cortex obtained from adult male albino rats after oral administration of ACMP, βC, and/or their combination (ACMP + βC) compared to control rats. (**a**) Control group; (**b**) βC-treated group; (**c**) ACMP-treated group; and (**d**) ACMP + βC group. G, Glomerulus; P, proximal convoluted tubule; D, distal convoluted tubule; stars show a decrease in the carbohydrate content of many renal tubules; βC, β-Carotene; ACMP, Acetamiprid; PAS, periodic acid-Schiff (Original magnification: a-d, X400)

Inspection of the control and βC groups revealed deep purple staining for polysaccharides in the renal tubules’ glomerulus, brush borders, and basement membrane (Figs. 7a and b, respectively). Compared to the control group, the ACMP group displayed marked depletion of PAS-reactivity, with many hepatocytes staining pale, indicating a lack of polysaccharides in the glomerulus and renal tubules (Fig. 7c). Sections from the ACMP + βC group showed an increase in polysaccharide content in the renal cortex compared to the ACMP group. The polysaccharide content in the ACMP + βC group appeared similar to that of the control group (Fig. 7d). Morphometric analysis of PAS reaction intensity in the ACMP group revealed a significant decrease (*p* < 0.05) compared with the control group. Furthermore, the morphometric analysis of PAS reaction intensity in the ACMP + βC group showed a significant increase (*p* < 0.001) compared with the ACMP group, with no significant difference observed compared to the control group (*p* > 0.05) (Table 4).

### MPO immunostaining of the kidney

**Fig. 8:**
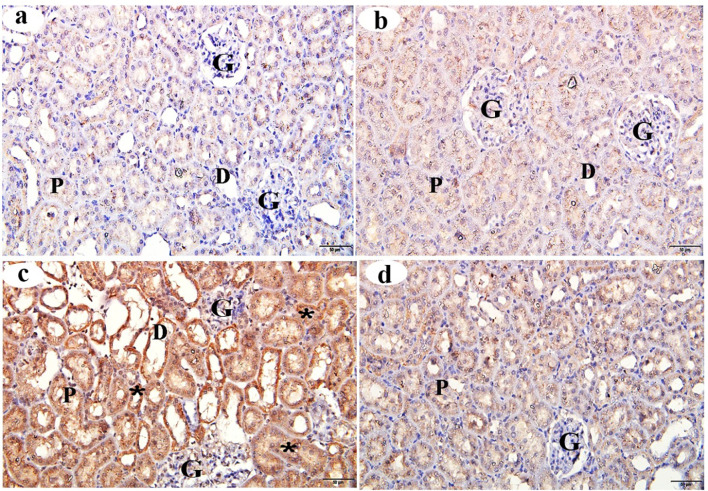
Immunohistochemical changes (stained with MPO immune-staining) of the renal cortex obtained from adult male albino rats after oral administration of ACMP, βC, and/or their combination (ACMP + βC) compared to control rats. (**a**) Control group; (**b**) βC-treated group; (**c**) ACMP-treated group; and (**d**) ACMP + βC group. G, Glomerulus; P, proximal convoluted tubule; D, distal convoluted tubule; stars show a strong positive MPO reaction in the many renal tubules; βC, β-Carotene; ACMP, Acetamiprid; MPO, myeloperoxidase (Original magnification: a-d, X400)

The cortical tissue sections of the control and βC groups revealed no MPO expression (Figs. 8a and b, respectively). In contrast, sections from the ACMP group showed strongly positive staining for MPO protein compared to the control group (Fig. 8c). A comparison between the ACMP + βC group and the ACMP group revealed that βC treatment was associated with a reduction in MPO-positive cells in the renal cortical tissues (Fig. 8d). Morphometric analysis of MPO intensity in the ACMP group showed a significant decrease (*p* < 0.001) compared to the control group. Additionally, the morphometric analysis of MPO intensity in the ACMP + βC group showed a significant reduction (*p* < 0.001) compared to the ACMP group (Table 4).

## Discussion

Acetamiprid is a type of neonicotinoid insecticide primarily used for pest control. However, like many chemicals, it can pose toxicity risks to non-target organisms, including humans, if not handled properly. In mammals, acetamiprid exhibits relatively low acute toxicity, but high doses can still produce adverse effects. Although acetamiprid has shown low hazard risks to humans under normal conditions, its potential for bioaccumulation necessitates careful dose selection. Studies have established acceptable daily intake levels based on observed effects in laboratory animals [26]. However, in cases of toxicity, materials with protective effects at safe doses should be tested for their ability to mitigate such toxicity. Beta-carotene has been shown to have a protective effect. The Joint FAO/WHO recommends an acceptable daily intake of beta-carotene of 0–5 mg/kg body weight, emphasizing the safety of lower doses for the general population [25].

The results of the present study indicated that the levels of TOS in liver and kidney tissues showed a highly significant increase, while the levels of TAC displayed a significant decrease in the ACMP group compared to the control group. These findings are consistent with previous studies that reported ACMP-treated rats experienced oxidative stress and lipid peroxidation, attributed to decreased TAC and antioxidant enzyme activity [9, 30–32]. Recent research stated that oxidative stress plays a key role in the mechanism of ACMP toxicity [5, 33], which is associated with a reduction in antioxidant enzyme activity [32, 34–37].

In the present study, ACMP induced several histological alterations in both hepatic and renal tissues. ACMP toxicity caused several deteriorative changes in the structure of these two vital organs, associated with an increase in collagen fiber content in both. Similarly, recent research reported marked histopathological abnormalities and increased collagenous fibers in liver and kidney sections exposed to ACMP [5, 9, 33]. Previous studies showed that the liver and kidneys are the primary targets of ACMP exposure due to its metabolism [4, 7, 32, 38]. The possible interpretation of the histopathological changes observed in the present study is related to the tissue injury and oxidative stress induced by ACMP, which results in tissue damage [39, 40]. Furthermore, congestion and reduced blood circulation caused by ACMP may lead to anoxia, resulting in tissue degeneration [40].

The histochemical results of this study confirmed that ACMP-induced depletion of PAS-reactivity occurred in numerous hepatocytes and nephrocytes. These findings are consistent with recent studies that reported decreased liver glycogen content following insecticide administration [41, 42]. Additionally, the damage extended to the kidneys, with severe depletion of polysaccharide content in renal cells being reported [43]. This depletion of glycogen content due to insecticide exposure may be interpreted as a result of decreased hepatic glucokinase activity, an enzyme essential for glycogen synthesis. This is consistent with the detrimental effects of the pesticide on glycogen content and glycogenesis [44].

The current study revealed that immunohistochemical staining of MPO showed marked MPO expression in the liver and kidney sections of the ACMP-treated group compared to the control group. A previous study observed that administering neonicotinoid insecticides increases MPO levels in liver tissues [45]. Mounting evidence suggests that oxidative stress and inflammatory responses are closely associated [46]. MPO is a catalytic agent in synthesizing hypochlorous acid, which has toxic effects on various cellular components, thereby increasing oxidative damage [47]. Moreover, the elevated level of MPO activity serves as one of the most reliable diagnostic markers for inflammation and oxidative stress in various commonly occurring disorders [48]. These findings support the observation in the current study that ACMP administration induces oxidative stress and leads to inflammation [49].

βC, a carotenoid pigment, functions mainly as pro-vitamin A in animals. It is a potent free radical scavenger and chain-breaking antioxidant [50]. The present study showed that the co-administration of βC in the ACMP + βC group modulates ACMP-toxic effects to bring them back to the normal range and causes improvements in all investigated parameters. The ACMP + βC group exhibited a significantly decreased level of TOS and a significantly higher level of TAC, associated with reduced degenerative changes in the liver and kidney. These results are consistent with previous findings that βC supplementation decreased free radical levels and increased antioxidant activities [51].

The findings of the antioxidative effect of βC in the present study can be attributed to the fact that βC is an efficient singlet oxygen quencher, preventing the formation of singlet oxygen by quenching excited triplet sensitizers [52]. The conjugated double bonds allow βC to accept electrons from reactive species, thereby neutralizing free radicals.

Similarly, the present study’s findings resemble those previously reported, confirming that βC exhibits protective activity against histological abnormalities and fibrosis in hepatic tissue [50, 53–55] and renal tissue of rats [25, 55–57]. It can be concluded that the protective effect of βC is related to its antioxidant, anti-inflammatory, and anti-apoptotic activities, as previously documented [58–60]. Moreover, previous studies have confirmed that βC can aid in restoring tissue glycogen and increase both hepatic and renal glycogen levels [23, 61, 62].

Evaluation of MPO activity in the βC-treatment group in the present study revealed a significant reduction in MPO activity, consistent with previous studies [19, 63]. In addition to its antioxidant effects, βC possesses anti-inflammatory properties, which lead to the inhibition of neutrophil infiltration, immune enhancement, and downregulation of key cytokines [64, 65]. These effects may help explain the reduction in MPO immunostaining, which is considered a marker of inflammation in hepatic and renal tissues.

## Conclusion

Administration of ACMP diminished antioxidants and caused oxidative stress and hepatic and renal histopathological, histochemical, and immunohistochemical alterations. The use of βC was found to reduce the harmful effects of ACMP across all the tested parameters. While the current findings suggest that βC administration may mitigate the harmful effects of ACMP in the studied rat model, further research, including studies on both sexes and human trials, is necessary to confirm its effectiveness and potential applications for farmers, agricultural workers, and consumers.

**Table 1:** General toxicity profile of Acetamiprid

General toxicity parameters	Data Summary
Mortality rate	No mortality observed.
Appetite	Decreased food intake.
Activity signs	Early stage: marked irritation and excitement.
Locomotion	Later stage: decreased motor activity.
Psychological signs	Later stage: tendency toward isolation and remaining in cage corners.
Body Strength	General weakness observed over time.
Skin Irritation	Slight irritation in the skin
Eye Irritation	Slight irritation to the eye
Specific Effects on Vital Organs	
Liver	Liver damage associated with the following changes in the hepatic tissue • Progressive degenerative changes • Significant increase in total oxidative stress biomarker. • Significant decrease in total antioxidant capacity biomarker
Kidney	kidney damage associated with the following changes in the renal tissue • Progressive degenerative changes • Significant increase in total oxidative stress biomarker. • Significant decrease in total antioxidant capacity biomarker

**Table 2:** Mean **±** SD of the total oxidative stress and total antioxidant capacity in liver and kidney tissue homogenates of the studied groups

		Control group	βC group	ACMP group	ACMP + βC group
Liver tissue	Total oxidative stress (mmol/g)	0.051 **±** 0.036	0.048 **±** 0.027	0.3194 **±** 0.216 ^a^	0.1003 **±** 0.045 ^b^
	Total antioxidant capacity (mmol/g)	1.203 ± 0.301	1.060 ± 0.493	0.5171 ± 0.161^a^	1.157 ± 0.445 ^b^
Kidney tissue	Total oxidative stress (mmol/g)	0.1821 ± 0.088	0.238 ± 0.063	0.526 ± 0.135 ^a^	0.322 ± 0.092 ^b^
	Total antioxidant capacity (mmol/g)	1.887 ± 0.645	1.690 ± 0.5220	0.561 ± 0.201 ^a^	1.334 ± 0.418 ^b^

SD: Standard deviation; ACMP: Acetamiprid, βC: β-carotene

^a^ Significantly different from the control group at *p* < 0. 05

^b^ Significantly different from the ACMP group at *p* < 0. 05

**Table 3:** Histopathological findings and their scores in selected organs among various studied groups

	Control group	βC group	ACMP group	ACMP + βC group)
**Liver**				
Congestion of the central veins and blood sinusoids	-	-	+++++	**±**
Hepatocytes with basophilic cytoplasm	-	-	+++++	**±**
Hepatocytes with vacuolar cytoplasm	-	-	+++++	**±**
Hepatocytes with pyknotic nuclei	-	-	+++++	**±**
**Kidneys**				
Atrophy of renal corpuscles	-	-	+++++	**±**
Reduction in glomerular cellularity	-	-	+++++	**±**
Dilation of the urinary space	-	-	+++++	**±**
Inflammatory cell infiltration in renal tissue	-	-	+++++	**±**
Congestion of interstitial blood capillaries	-	-	+++++	**±**

ACMP: Acetamiprid, βC: β-carotene

(-) the change was not found

(+++++) the change was very often found in all animals

(±) the change was sporadic

**Table 4:** Mean **±** SD of the of morphometric analysis of the % intensity of PAS reaction, collagenous fibers and MPO immunostaining / surface area of both liver and kidney sections

	Control Group	βC group	ACMP group	ACMP + βC group)
*Liver tissue*				
% Intensity collagenous fibers/surface area	9.434 ± 1.048	7.206 ± 0.9627	22.65 ± 1.979 ^a^	15.57 ± 0.8494 ^b^
% Intensity of PAS reaction/surface area	50.33 ± 2.873	44.57 ± 5.012	25.23 ± 3.011 ^a^	40.13 ± 1.650 ^b^
% Intensity MPO/ surface area	8.633 ± 1.030	6.677 ± 0.5941	23.85 ± 1.727 ^a^	11.73 ± 0.7979 ^b^
*Kidney tissue*				
% Intensity collagenous fibers/surface area	10.94 ± 0.4041	9.240 ± 0.8711	22.80 ± 2.241 ^a^	14.22 ± 1.535 ^b^
%Intensity of PAS reaction/surface area	106.1 ± 2.639	105.5 ± 4.854	95.58 ± 2.327 ^a^	112.3 ± 1.081^b^
% Intensity MPO/ surface area	8.396 ± 0.5219	7.296 ± 0.5426	20.58 ± 1.135 ^a^	11.47 ± 0.9115 ^b^

SD: Standard deviation; ACMP: Acetamiprid, βC: β-carotene

^a^ Significantly different from the control group at *p* < 0.05

^b^ Significantly different from the ACMP group at *p* < 0. 05

## Data Availability

This article contains all the data that was created or evaluated during the research.

## Abbreviations

ACMP: Acetamiprid
βC: β-carotene
TAC: Total antioxidant capacity
TOS: Total oxidative stress
